# Identification and validation of inferior prognostic genes associated with immune signatures and chemotherapy outcome in acute myeloid leukemia

**DOI:** 10.18632/aging.203166

**Published:** 2021-06-18

**Authors:** Jie Wang, Jian-Ping Hao, Md. Nazim Uddin, Yun Wu, Rong Chen, Dong-feng Li, Dai-Qin Xiong, Nan Ding, Jian-Hua Yang, Xuan-Sheng Ding

**Affiliations:** 1 School of Basic Medicine and Clinical Pharmacy, China Pharmaceutical University, Nanjing 211198, China; 2 Department of Hematology, First Affiliated Hospital of Xinjiang Medical University, Urumqi 830011, China; 3 Department of General Medicine, First Affiliated Hospital of Xinjiang Medical University, Urumqi 830011, China; 4 Department of Pharmacy, First Affiliated Hospital of Xinjiang Medical University, Urumqi 830011, China

**Keywords:** acute myeloid leukaemia, weighted gene co-expression network analysis, key genes, survival prognosis, immune infiltration

## Abstract

Acute myeloid leukemia (AML) is a group of heterogeneous hematological malignancies. We identified key genes as *ITGAM* and lncRNA *ITGB2-AS1* through different bioinformatics tools. Furthermore, qPCR was performed to verify the expression level of essential genes in clinical samples. Retrospective research on 179 AML cases was used to investigate the relationship between the expression of *ITGAM* and the characteristics of AML. The critical gene relationship with immune infiltration in AML was estimated. The clinical validation and prognostic investigation showed that *ITGAM*, *PPBP*, and *ITGB2-AS1* are highly expressed in AML (*P* < 0.001) and significantly associated with the overall survival in AML. Moreover, the retrospective research on 179 clinical cases showed that positive expression of *ITGAM* is substantially related to AML classification (*P* < 0.001), higher count of white blood cells (*P* < 0.01), and poor chemotherapy outcome (*P* < 0.05). Furthermore, based on grouping *ITGAM* as the high and low expression in TCGA-LAML profile, we found that genes in the highly expressed *ITGAM* group are mainly involved in immune infiltration and inflammation-related signaling pathways. Finally, we discovered that the expression level of *ITGAM* and lncRNA *ITGB2-AS1* are not just closely related to the immune score and stromal score (*P* < 0.001) but also significantly positively correlated with various Immune signatures in AML (*P* < 0.001), indicating the association of these genes with immunosuppression in AML. The prediction of candidate drugs indicated that certain immunosuppressive drugs have potential therapeutic effects for AML. The critical genes could be used as potential biomarkers to evaluate the survival and prognosis of AML.

## INTRODUCTION

Acute myeloid leukemia (AML) is a highly heterogeneous hematological malignancy that seriously harms human health, and it is also the most common type of adult acute leukemia. The incidence of AML is about 3.7/100000, but the disease is progressing rapidly, and the age-related mortality is about 2.7/100000 to 18/100000 [1]. With the rapid development of microarray gene chip and NGS high-throughput sequencing technology, it is possible to accurately predict patients' diagnosis and prognosis with acute myeloid leukemia and provide an objective basis for more effective individualized treatment of leukemia [2]. In recent years, with the extensive research on the pathogenesis of AML, it has been confirmed that a variety of abnormal gene expressions may play an essential role in the occurrence and development of AML [3, 4].

Bioinformatics analysis is a method that is increasingly used to explore target genes and proteins. Weighted gene co-expression network analysis (WGCNA) is a systematic biological method to describe the association patterns between genes in micro-array sequencing samples. It can identify highly related gene clusters (modules) to study potential functions [5]. Recent studies have shown that WGCNA has been widely used to screen and identify AML susceptibility genes and candidate targets [6].

In the past two decades, many biological information data databases have been widely used in tumor-related research. The Gene expression Omnibus (GEO) database is a public bioinformatics database provided by the National Center for Biotechnology Information (NCBI) to store, query, and download the gene chip results NGS and another high-throughput sequencing. It is one of the largest gene chip databases in the world [7]. The Cancer Genome Atlas (TCGA) database is a comprehensive multi-dimensional map of fundamental genomic changes in 33 cancers, jointly provided by The National Cancer Institute (NCI) and The National Human Genome Research Institute (NHGRI). It can give the researchers comprehensive cancer genome data sets related to tumor stage, metastasis, survival rate, patient information, and so on [8]. In recent years, many research centers have developed online visual analysis databases for TCGA tumor-related information. The online databases such as GEPIA have processed RNA sequencing expression data from 9736 tumors and 8587 standard samples from TCGA and GTEx projects. And the online database can further achieve according to different tumor types or pathological stages of survival analysis, similar gene detection, correlation analysis, and other functions [9].

The tumor microenvironment (TME) is the cellular environment in which tumor lesions exist [10]. It comprises immune cells, stromal cells, endothelial progenitor cells, extracellular matrix, growth factors, and cytokines [11]. In recent years, studies have found that the bone marrow microenvironment plays a vital role in the homing and survival of leukemia cells [12, 13]. Besides, the interaction between leukemia cells and the bone marrow microenvironment affects the survival of acute myeloid leukemia cells [14, 15]. It also has a specific impact on chemotherapy drugs' sensitivity and resistance [16, 17]. At present, the ESTIMATE program is a beneficial method to explore the microenvironment in many tumors [18]. It has been used to investigate the prognostic genes in the microenvironment of AML patients [19].

In this study, we utilized the transcriptome sequencing data of adult AML to identify the differentially expressed genes and construct the co-expression network. And that we have screened the essential genes that significantly affect the survival and prognosis of AML. Besides, we have validated the expression of critical genes in clinical samples, investigating the relationship between the expression of essential genes and patients' characteristics in retrospective research. Furthermore, we have predicted the potential drugs targeting critical genes and the impact of essential genes on the immune infiltration of AML.

## RESULTS

### Identification of differentially expressed genes related to acute myeloid leukemia

**Figure 1:**
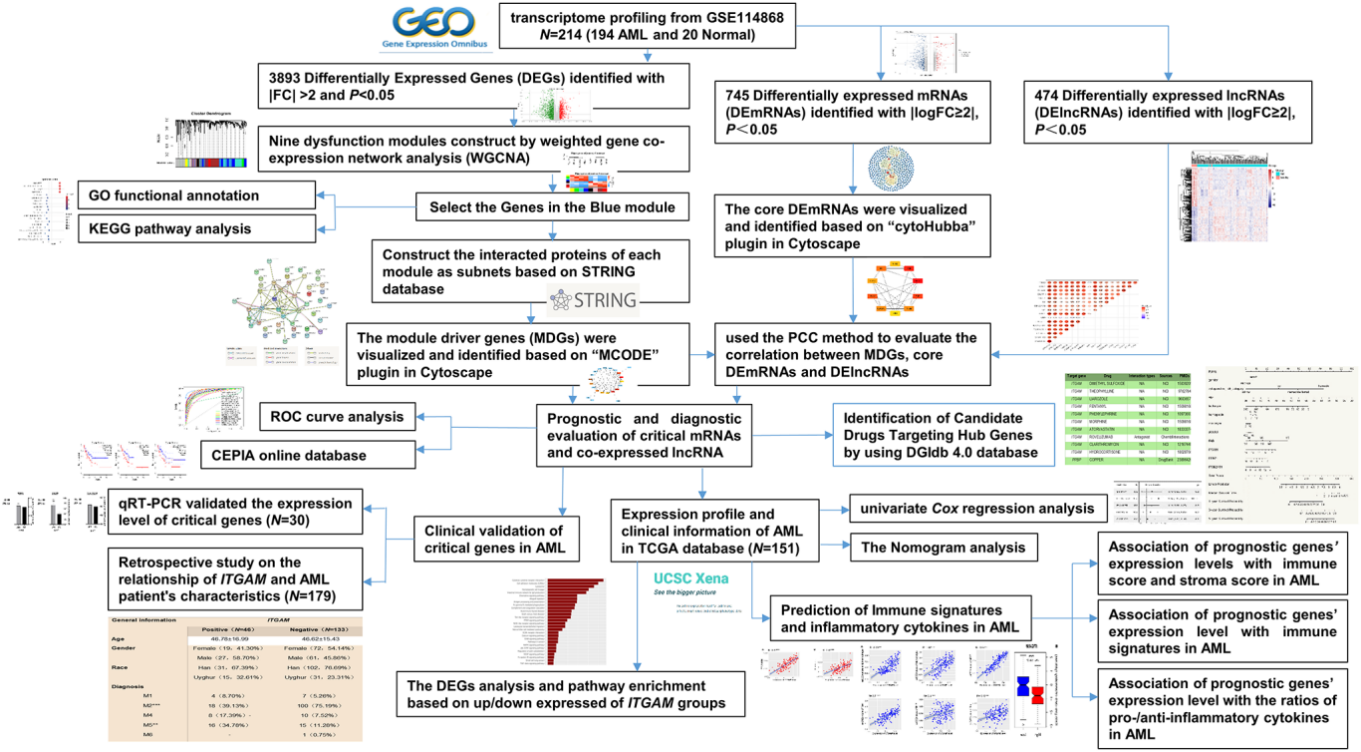
The overall analysis process of the present study.

**Figure 2:**
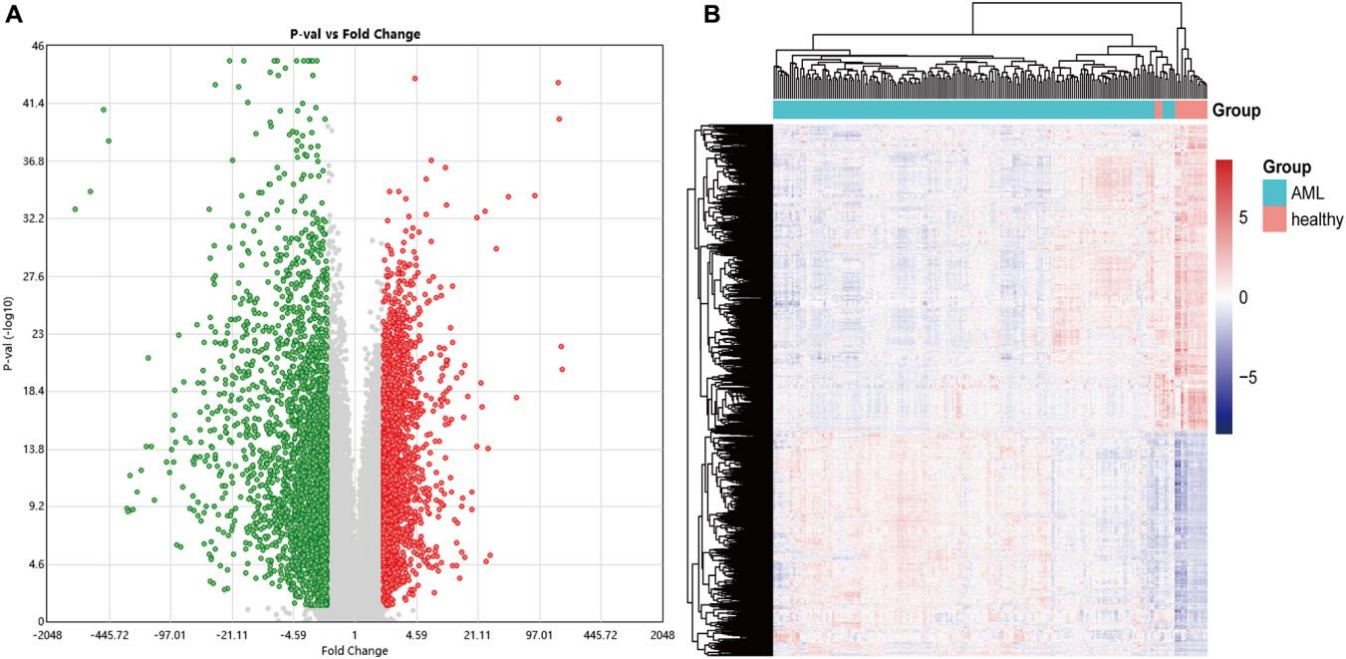
**The volcano and heatmap of the differentially expressed genes.** (**A**) In the DEGs' volcano plot, red dots are up-regulated genes, blue is down-regulated, and grey is no different; (**B**) DEGs clustering heatmap, pink indicates healthy, and blue indicates AML. DEGs: differentially expressed genes.

This study obtained an expression profile data matrix that includes 67758 genes in 214 samples, the R software to process the GSE114868 transcriptome data. The workflow of the analysis procedure in this study was shown in Figure 1. Based on the screening criteria *as* |*FC*| > 2 and *P* < 0.05, 3893 differentially expressed genes (DEGs) were identified in total. These DEGs include 1657 up-regulated genes and 2236 down-regulated genes. We used R software to draw the volcano plot and heatmap of differentially expressed genes (Figure 2).

### Weighted co-expression network construction and identification of the critical module

**Figure 3:**
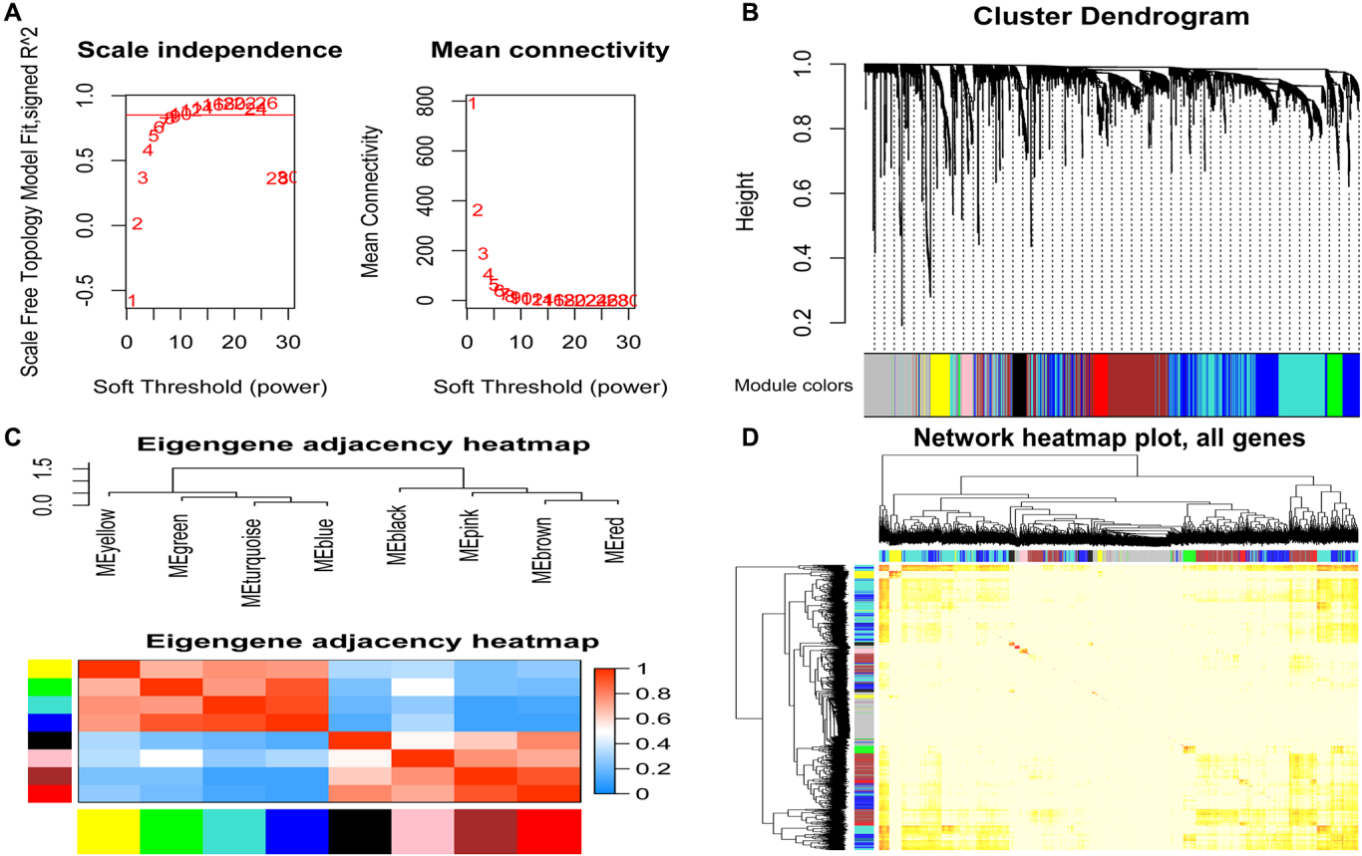
**Identification of modules related to clinical features of acute myeloid leukemia.** (**A**) Analysis of the soft threshold (β) through the scale-free fitting index and mean connectivity; (**B**) Clustering dendrogram of the DEGs through dissimilarity coefficient, which shows nine gene co-expression modules AML. Gray modules indicate no co-expression between genes; (**C**) The correlation heat map of WGCNA adjacent modules. The rectangles in each row and each column represent a module characteristic gene. Light blue represents low adjacency, and red represents high adjacency; (**D**) The TOM visualized the gene co-expression network's heat map in the module. In the TOM map, light colors indicate topological overlap. Dark colors indicate a higher degree of topological overlap. The gene tree diagram and corresponding modules are displayed on the upper left of the TOM diagram. The intersection of the two rectangles indicates the topological overlap in the Blue module. DEGs: differentially expressed genes; TOM: topological overlap matrix.

To find the co-expressed gene modules (Module), explore the relationship between the gene network and the phenotype of interest, and the core genes in the network, we used the WGCNA method to construct a weighted gene co-expression network. In the process of network construction, we selected the soft threshold *β* = 8 (*R*^2^ = 0.85) to establish a topological matrix whose gene distribution conforms to the scale-free network. The topological matrix was clustered with co-expressed genes through the coefficient of dissimilarity between genes. The gene co-expression network was divided into nine modules, and the topological overlap of adjacent modules was used to select characteristic functional modules. Finally, it determined that the Blue module has the highest module importance and containing 75 genes (Figure 3).

### Gene ontology (GO) functional annotation and Kyoto Encyclopedia of Genes and Genomes (KEGG) pathway enrichment analysis

**Figure 4:**
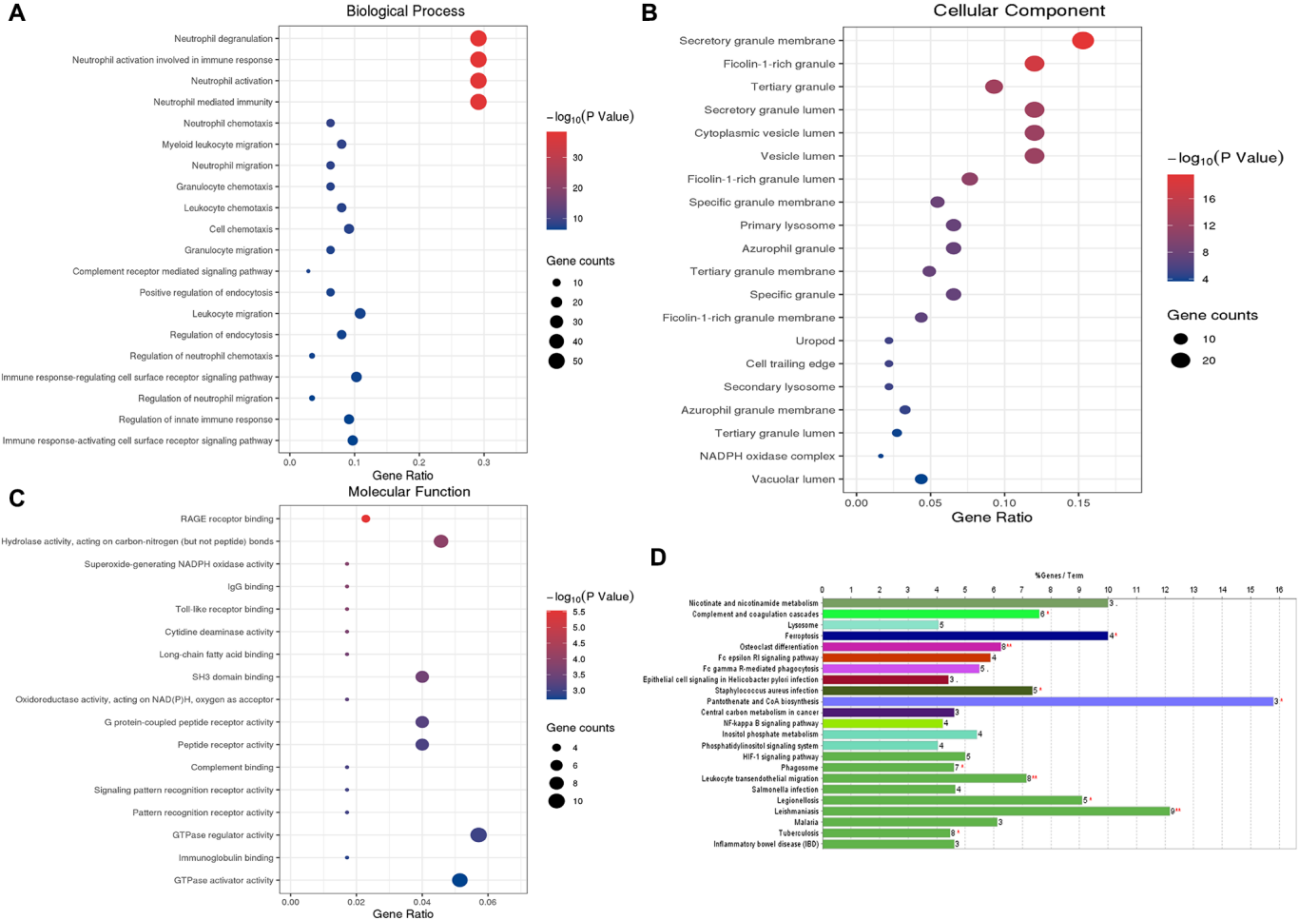
**GO functional enrichment analysis and KEGG pathway analysis of characteristic genes in the Blue module.** (**A**) Top 20 enriched biological process terms; (**B**) Top 20 enriched cell component enrichment; (**C**) The results of Molecular function enrichment analysis; (**D**) The results of the KEGG pathway enrichment analysis. Abbreviations: GO: gene ontology; KEGG: Kyoto Encyclopedia of Genes and Genomes.

We performed the GO functional annotations on 75 characteristic genes in the Blue module, including biological process (BP), Cellular Component (CC), and Molecular Function (MF) analysis. BP analysis showed that the co-expressed genes were mainly annotated in neutrophil degranulation, neutrophil activation, and neutrophils participating in the immune activation response. CC analysis showed that the co-expressed genes were significantly annotated in the cell components such as secretory granule membrane, ficolin granule, secretory granule lumen, and cytoplasmic cyst. MF analysis shows that the co-expressed genes are mainly related to carbon-nitrogen bond hydrolase activity, SH3-domain binding, and RAGE receptor binding. KEGG pathway enrichment analysis results show that the co-expressed genes mainly involve neutrophil migration, tumor carbon metabolism, iron death, FcγR-mediated phagocytosis, HIF-1, and NF-κB signaling pathway. These results indicate that the co-expressed genes in the necessary modules are mainly involved in cellular immune and inflammatory processes (Figure 4).

### Recognition of module driver genes based on PPI network

**Figure 5:**
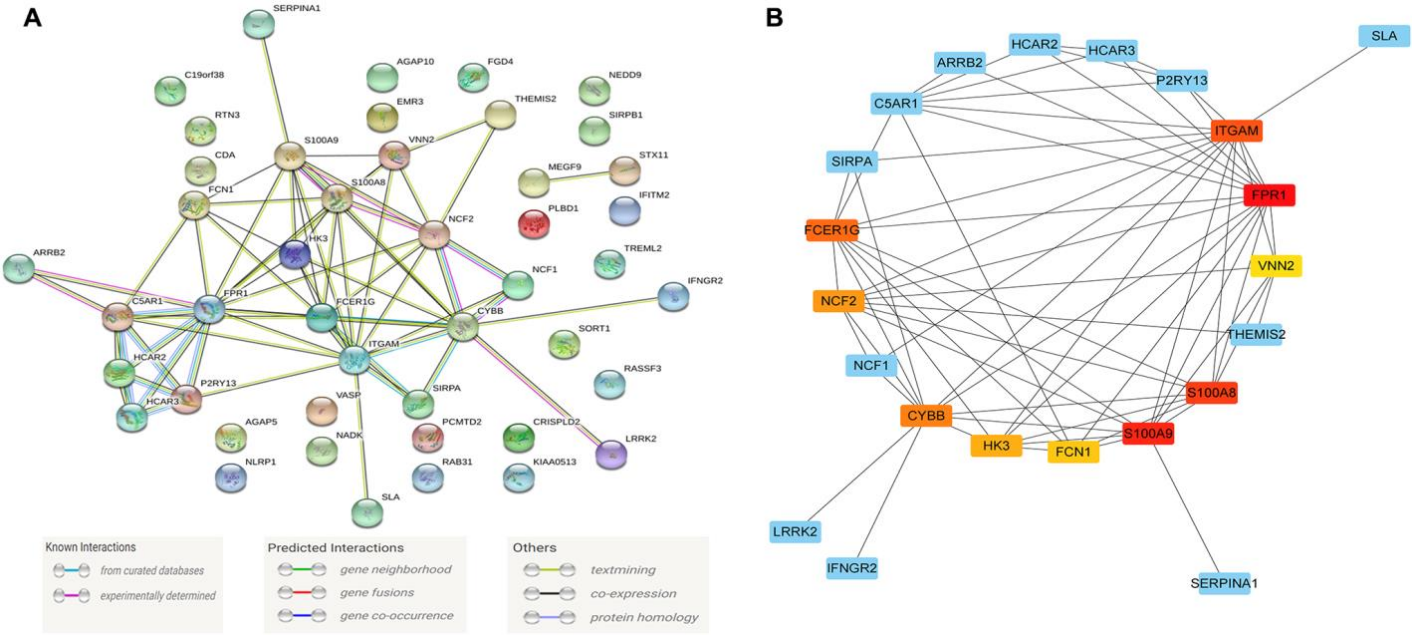
**The module driver genes identified in the PPI network.** (**A**) The PPI network consists of 45 nodes and 66 edges, and 75 of the Blue module's co-expression of genes constructs a PPI network; (**B**) Clusters of driver genes in the Blue module, and the squares marked in yellow to red indicate the top 10 module driver genes in sub-function cluster 1, and the blue squares represented the other related genes in cluster 1. Abbreviation: PPI: protein-protein interaction.

To further study the function of the characteristic genes in the Blue module at the protein level after the WGCNA analysis, we used the STRING database (https://string-db.org/) to screen 75 of the Blue modules co-expression of genes constructs a protein-protein interaction (PPI) network (Figure 5A).

The PPI network consists of 45 nodes and 66 edges, and we used the "MCODE" program in Cytoscape software to further identify the module driver genes (MDGs) in the PPI network. The cut-off value is set as K-core = 2. Finally, two functional clusters were screened out. Cluster 1 contains 10 MDGs (*S100A9, S100A8, NCF2, CYBB, ITGAM, FCER1G, FPR1, FCN1, HK3, VNN2*) (Figure 5B), and cluster 2 includes 4 MDGs (*HCAR3, HCAR2, C5AR1, P2RY13*) (Table 1).

**Table 1:** The cluster calculated by MCODE score in PPI network.

**MCODE_Cluster**	**MCODE_Score**	**Gene Name**
Cluster 1	6	*NCF2*
Cluster 1	6	*HK3*
Cluster 1	5.785714	*S100A9*
Cluster 1	5.785714	*S100A8*
Cluster 1	5.785714	*CYBB*
Cluster 1	5.785714	*ITGAM*
Cluster 1	5.785714	*FCER1G*
Cluster 1	5.785714	*FPR1*
Cluster 1	5	*FCN1*
Cluster 1	5	*VNN2*
Cluster 2	4	*HCAR3*
Cluster 2	4	*HCAR2*
Cluster 2	4	*C5AR1*
Cluster 2	4	*P2RY13*

### Identification of core differentially expressed mRNAs (DEmRNAs) in the whole transcriptome

**Figure 6:**
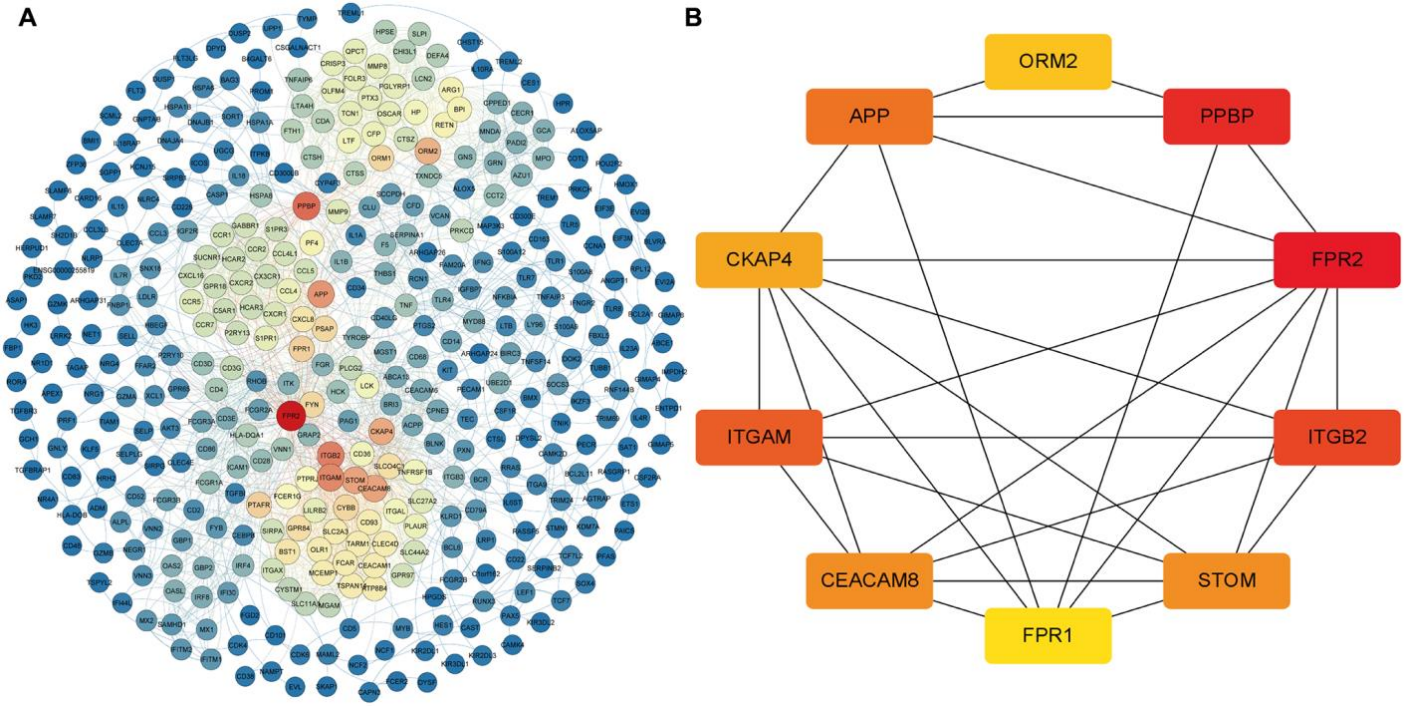
**The core DEmRNAs identified in the PPI network.** (**A**) The darker the color (red) of the genes, the higher degree of contribution in the PPI network; (**B**) The darker color (red) of the DEmRNA represents the gene with a higher degree. Abbreviations: DEmRNAs, differentially expressed lncRNAs. PPI: protein-protein interaction.

To further verify the regulatory function of the obtained MDGs in the whole transcriptome, we also identified 745 DEmRNAs in the transcriptome data with more stringent screening conditions (|*logFC* ≥ 2|, *P* < 0.05), and using the STRING database constructed a PPI network, which contains 392 nodes and 2357 edges (Figure 6A). Besides, we also use the "cytoHubba" program in Cytoscape software to calculate the degree of contribution (Degree) in the PPI network, then obtain the ten core DEmRNAs (*FPR2, PPBP, ITGB2, ITGAM, APP, CEACAM8, STOM, CKAP4, ORM2, FPR1*) with the highest degree of contribution (Degree) in the global network (Figure 6B). Then the obtained top 10 DEmRNAs are compared with the MDGs in the module (Blue). The results show that *ITGAM* and *FPR1* are common genes, and these two common genes show vital global regulatory functions. They may play an essential role in the pathogenesis of AML.

### Screening and correlation analysis of differentially expressed lncRNAs (DElncRNAs)

**Figure 7:**
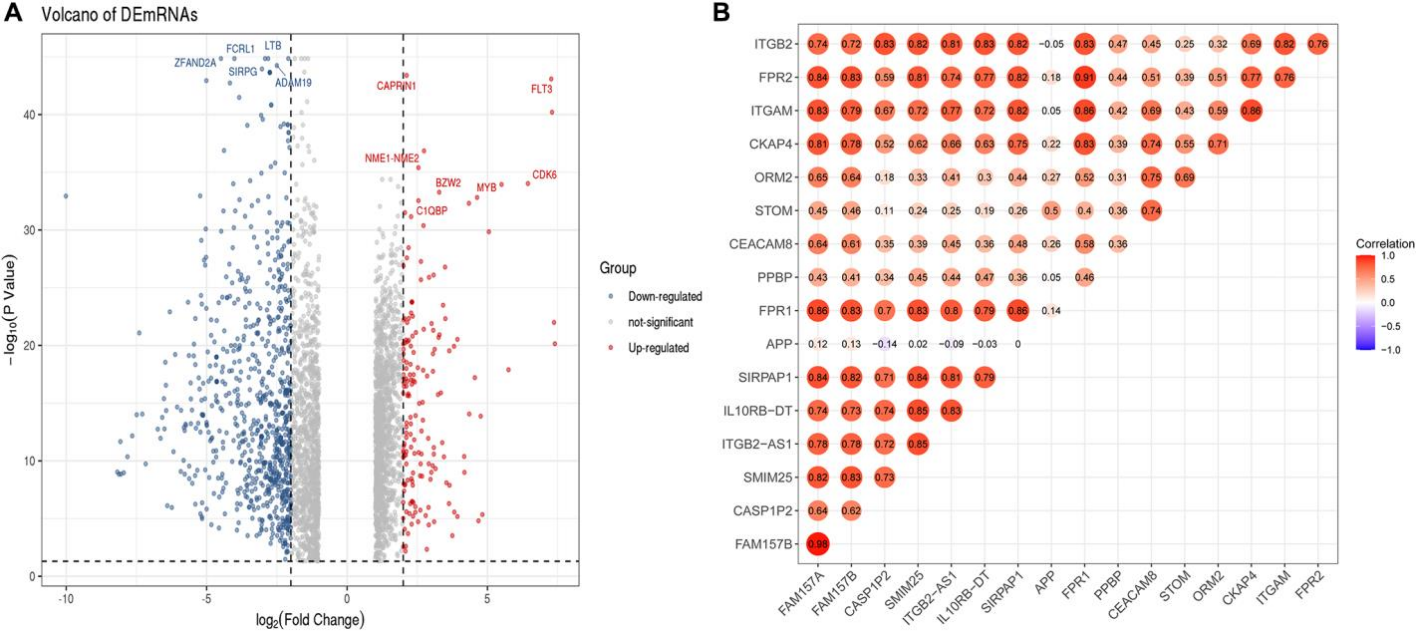
**Identification of DElncRNAs and the co-relationship of core DEmRNAs and DElncRNAs.** (**A**) In the volcano plot of the DElncRNAs, red dots are up-regulated lncRNAs, blue is down-regulated, and grey is no different; (**B**) The expression relevance of DEmRNAs and DElncRNAs. The darker the color (red) of the circle, the stronger the correlation. Abbreviations: DEmRNAs: differentially expressed mRNAs; DElncRNAs: differentially expressed lncRNAs.

To further determine the potential regulatory relationship between differentially expressed lncRNAs (DElncRNAs) and core DEmRNAs in AML and clarify the regulatory function of DElncRNAs. We screened 474 DElncRNAs from the transcriptome data and displayed the volcano plot of DElncRNAs by the R software (Figure 7A).

After that, we used the PCC method to evaluate the correlation between core DEmRNAs and DElncRNAs, then screened the noteworthy DElncRNA-mRNA pairs (*PCC* > 0.8, *P* < 0.05). The results showed that the expression of 7 lncRNAs (*SIRPAP1, ITGB2-AS1, FAM157B, FAM157A, CASP1P2, SMIM25, IL10RB-DT*) significantly correlated with the expression of core mRNAs (Figure 7B). Among them, the expression of DElncRNAs includes *FAM157A* and *SIRPAP1* that are mainly related to the expression of *ITGAM*. Furthermore, DElncRNAs as *FAM157A, FAM157B, SMIM25, ITGB2-AS1*, and *SIRPAP1* are significantly associated with the expression of *FPR1*.

### Prognostic evaluation of critical mRNAs and co-expressed lncRNA

To verify the prognostic value of the screened essential genes and co-expressed DElncRNAs, we conducted prognostic verification on the 18 important genes (including two common genes) and 7 lncRNAs selected based on the TCGA-LAML clinical data in the GEPIA online database (http://gepia2.cancer-pku.cn).

**Figure 8:**
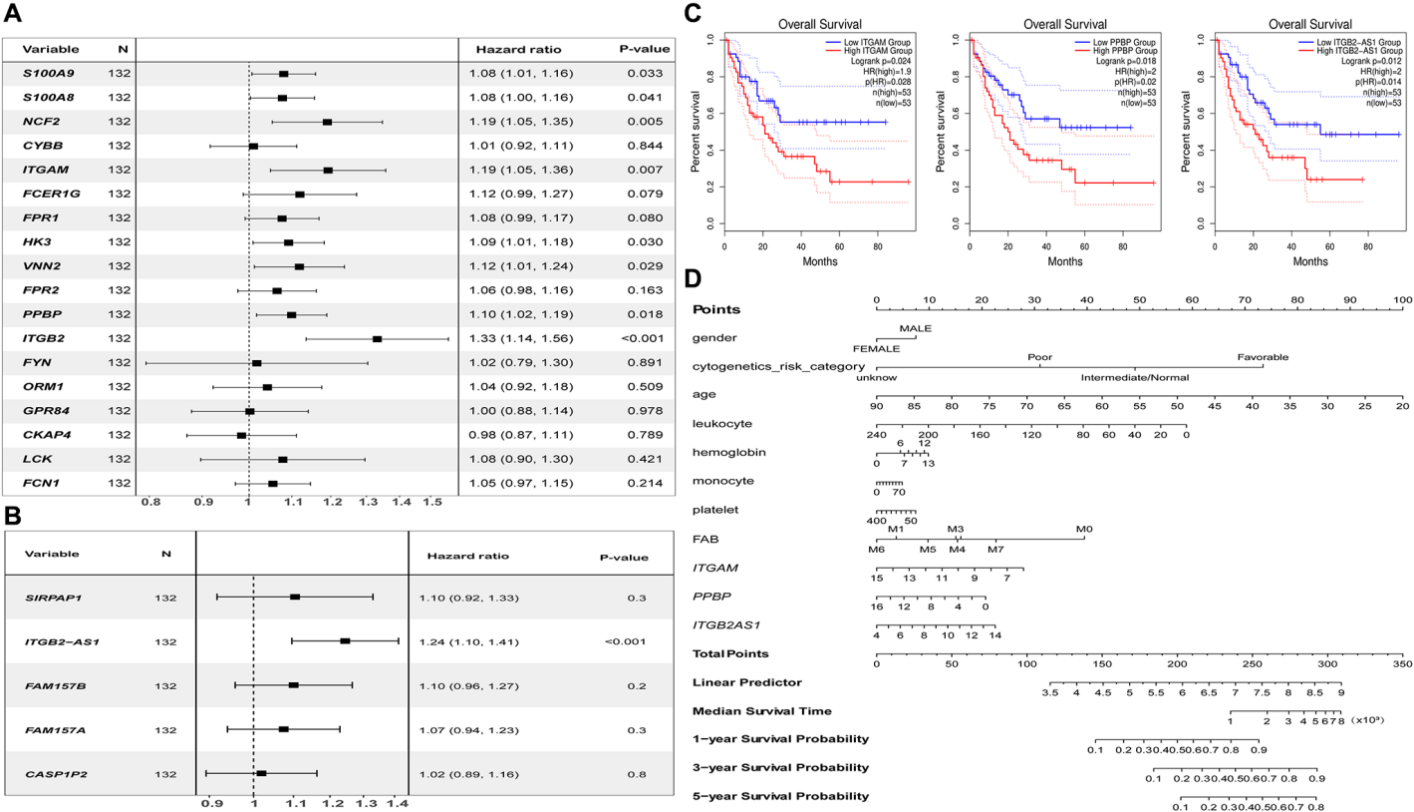
**Prognostic analysis results of essential mRNAs and co-expressed DElncRNAs.** (**A**) The result of univariate Cox regression analysis showed that the key genes such as *S100A8* (HR:1.119, 95% CI:1.01–1.16)*, S100A9* (HR:1.08, 95% CI:1.00–1.16)*, NCF2* (HR:1.19, 95% CI:1.05–1.35)*, ITGAM* (HR:1.19, 95% CI:1.05–1.36)*, HK3* (HR:1.09, 95% CI:1.01–1.08)*, VNN2* (HR:1.12, 95% CI:1.01–1.24)*, PPBP* (HR:1.10, 95% CI:1.02–1.19), and *ITGB2* (HR:1.33, 95% CI:1.14–1.56) both have significant impact on the prognosis of AML patients (*P* < 0.05). (**B**) The development of the research showed that the expression of DElnRNAs as *ITGB2-AS1* (HR:1.24, 95% CI:1.10–1.41) has a significant impact on the prognosis of AML patients (*P* < 0.05). (**C**) The results of K–M survival analysis showed that AML patients with high expression of *ITGAM, PPBP*, and *ITGB2-AS1* had a poor prognosis (*P* < 0.05). (**D**) The Nomogram was established based on the clinical information of TCGA-LAML. The points for 11 factors (gender, cytogenetics risk category, age, leukocyte, hemoglobin, monocyte, platelet, FAB classification, and the expression level of *ITGAM*, *PPBP*, or *ITGB2-AS1*) were listed in the Nomogram. The score for each factor in the Nomogram was read out by drawing a straight line from the predictor to the point axis, and then the survival rates of 1, 3, and 5 years could be estimated by adding the points corresponding to each factor in the bottom scale. Abbreviations: DElncRNAs: differentially expressed lncRNAs; HR: hazard ratio; CI: confidence interval.

The univariate Cox regression analysis showed that both the critical genes as *S100A9, S100A8, NCF2, ITGAM, HK3, VNN2, PPBP, ITGB2* (Figure 8A), and co-expressed DElncRNA as *ITGB2-AS1* (Figure 8B) are the risk factors affecting the prognosis of AML patients (*P* < 0.05). The results of K-M survival analysis showed that AML patients with high expression of *ITGAM, PPBP*, and *ITGB2-AS1* had a poor prognosis (*P* < 0.05). In contrast, the common gene expression as *FPR1* did not significantly affect AML patients' prognosis. It is worth noting that, combining the results of the two prognostic analyses, we found that *ITGAM, PPBP*, and *ITGB2-AS1* both significantly impact AML patients' prognosis (Figure 8C). Finally, The Nomogram (Figure 8D) shows the probability of these three genes influencing the prognostic outcome.

### Quantitative real-time PCR (qRT-PCR) validated the expression level of critical genes in initial diagnosis patients with AML

**Figure 9:**
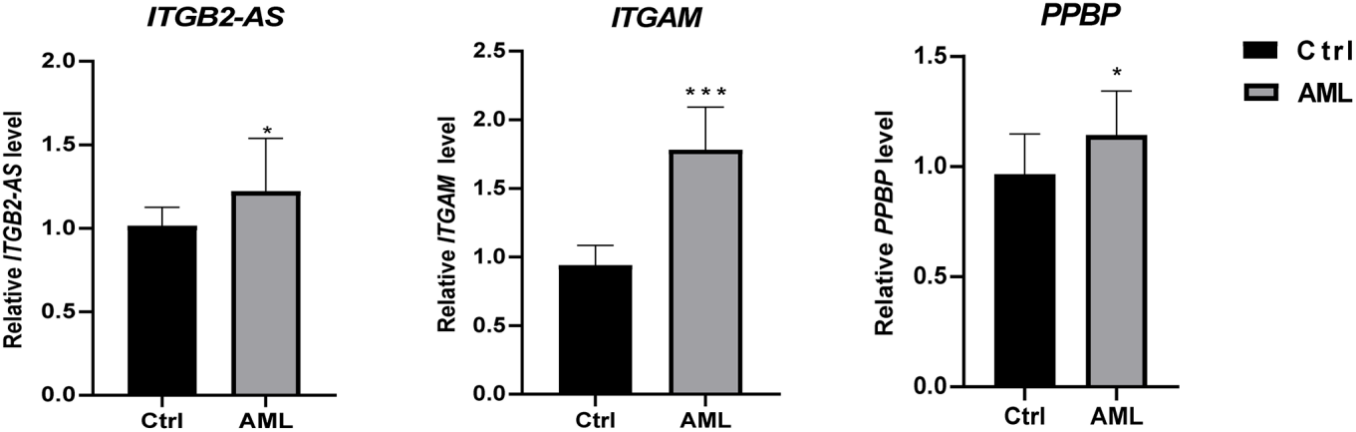
**The differential expression of critical genes in clinical samples between AML and healthy individuals.** The result of clinical validation showed that *ITGB2-AS, ITGAM,* and *PPBP* are significantly higher in the initial diagnosed AML. ^***^P < 0.001; ^*^P < 0.05.

We used 20 clinical samples to verify the different expression levels of essential genes between AML (15 bone marrow samples from initial diagnosis of AML) and health group (15 peripheral blood samples from anonymized healthy individuals). The validation result showed that *ITGB2-AS1,* *ITGAM,* and *PPBP* were significantly higher expressed in the AML group (Figure 9).

### The ROC curve analysis of the diagnostic value between MDGs, core DEmRNAs and co-expressed DElncRNAs

**Figure 10:**
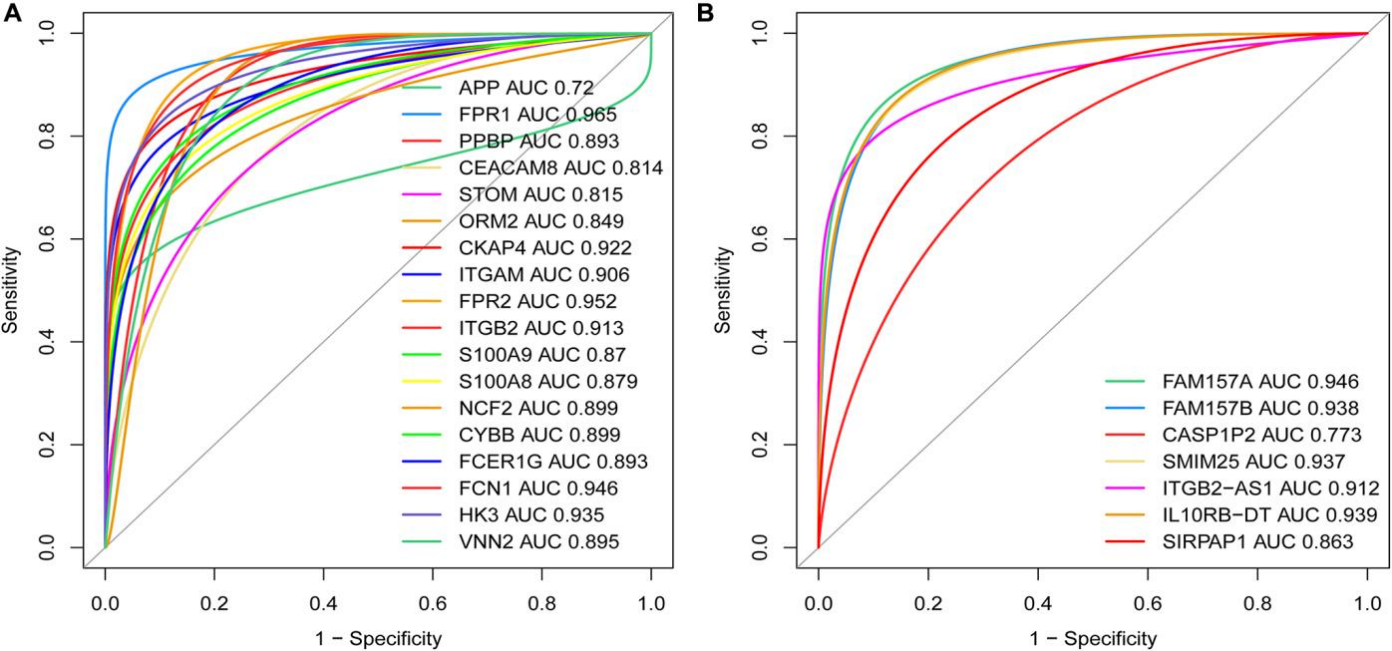
**Weights of the key mRNAs and co-expressed DElncRNAs were determined by the AUC values of the ROC curves in AML.** (**A**) ROC analysis revealed that the AUC for 18 mRNAs was ≥ 0.7; (**B**) ROC curve analysis shows that the AUC for 7 co-expressed DElncRNAs was also ≥ 0.7. Abbreviations: ROC: receiver operating characteristic; AUC: area under the curve; DElncRNAs: differentially expressed lncRNAs.

To further verify the diagnostic value between MDGs, core DEmRNAs and co-expressed DElncRNAs in AML, we used receiver operating characteristic (ROC) curve analysis to evaluate these genes. The study results showed that the area under the curve (AUC) of MDGs, core DEmRNAs and co-expressed DElncRNAs were both ≥ 0.7 (Figure 10), indicating that the critical genes that were screened in this study are good diagnostic value for AML.

### Retrospective research about the relationship between the expression of *ITGAM* and AML patient's characteristics

To understand the correlation between the expression level of *ITGAM* and the clinical characteristics of AML patients, we also investigated the clinical data of 179 AML patients and analyzing the impact of *ITGAM* expression on patient's features through a retrospective study. We have reported the general information of AML patients with differentially expressed *ITGAM* in Table 2. The study results show that the positive expression of *ITGAM* is substantially related to AML classification (*P* < 0.001) and a higher count of white blood cells (*P* < 0.01). It is noteworthy that the complete response (CR) rate of AML patients, received primary chemotherapy with negative expression of *ITGAM* was significantly higher than the positive expression of *ITGAM* (*P* < 0.05). Still, there was no remarkable difference in the no remission (NR) rate or the partial remission (PR) between the two groups.

**Table 2:** The general information distinguished by *ITGAM* expression and the characteristics of AML patients.

**General information**	***ITGAM***	
	**Positive (*N* = 46)**	**Negative (*N* = 133)**
*Age*	46.78 ± 16.99	46.62 ± 15.43
*Gender*	Female (19, 41.30%) Male (27, 58.70%)	Female (72, 54.14%) Male (61, 45.86%)
*Race*	Han (31, 67.39%) Uyghur (15, 32.61%)	Han (102, 76.69%) Uyghur (31, 23.31%)
WBC^**^	62.99 ± 71.05	34.81 ± 59.55
*Hb*	81.28 ± 22.88	80.99 ± 22.80
*PLT*	58.59 ± 57.76	79.71 ± 153.08
**Diagnosis**		
M1	4 (8.70%)	7 (5.26%)
M2^***^	18 (39.13%)	100 (75.19%)
M4	8 (17.39%)-	10 (7.52%)
M5^**^	16 (34.78%)	15 (11.28%)
M6	–	1 (0.75%)
**Prognostic generate**		
*WT-1*	33 (71.74%)	94 (70.68%)
*ASXL1*	13 (28.26%)	25 (18.80%)
*TET-2*	11 (23.91%)	22 (16.54%)
*DNMT3A*	6 (13.04%)	13 (9.77%)
*CEBPA*	4 (8.70%)	12 (9.02%)
*TP53*	6 (13.04%)	5 (3.76%)
*NPM1*	4 (8.70%)	15 (11.28%)
*FLT3*	4 (8.70%)	13 (9.77%)
*IDH1/IDH2*	0/3 (6.52%)	3 (2.26%)/2 (1.50%)
*C-kit*	3 (6.52%)	10 (7.52%)
*RUNX1*	2 (4.35%)	3 (2.26%)
*NRAS*	2 (4.35%)	3 (2.26%)
**Regimens**		
Un-Treatment	8 (17.39%)	11 (8.15%)
IA	23 (50.00%)	73 (54.89%)
DA	9 (19.57%)	38 (28.57%)
CAG	4 (8.70%)	3 (2.22%)
HMAs	2 (4.35%)	8 (5.93%)
**Treatment outcomes**	**(*N* = 38)**	**(*N* = 122)**
CR^*^	20 (52.63%)	90 (73.77%)
PR	4 (10.53%)	5 (4.10%)
NR	8 (21.05%)	21 (17.21%)
Death	6 (15.79%)	6 (4.92%)

^***^P < 0.001; ^**^P < 0.01; ^*^P < 0.05. Abbreviations: CR: complete remission; PR: partial remission; NR: no response; WBC: white blood cells; Hb: hemoglobin; PLT: platelet; IA: idarubicin, cytarabine; DA: daunorubicin, cytarabine; CAG: cytarabine, aclacinomycin, and granulocyte colony-stimulating factor; HMAs: hypomethylating agents mainly contain azacytidine or decitabine.

### Pathway analysis based on grouping *ITGAM* as high and low expression in TCGA-LAML

**Figure 11:**
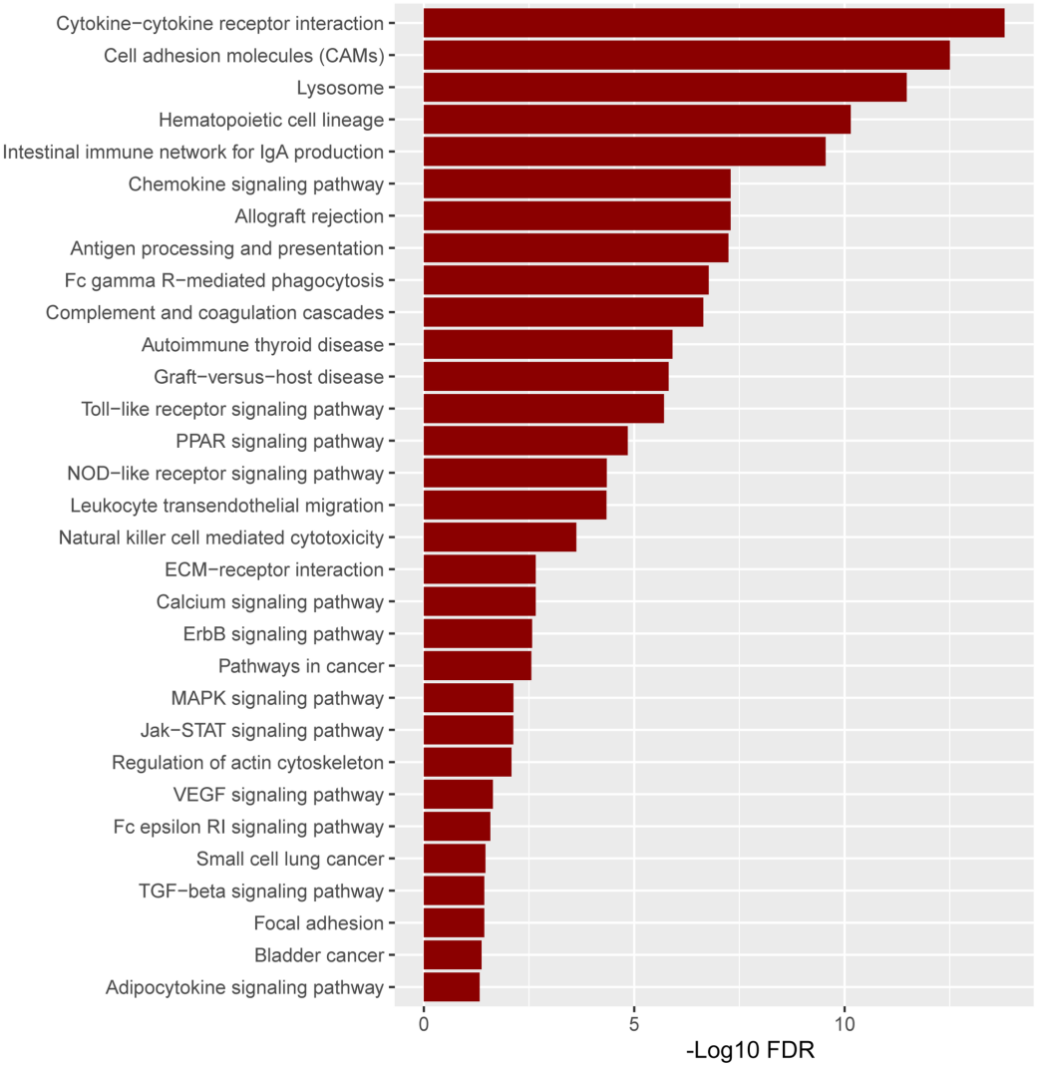
**The pathway enrichment in the high versus low *ITGAM* expression group is based on the TCGA-LAML expression profiles.** The significant enriched KEGG pathways were confirmed as an enrichment when FDR < 0.05.

Based on the substantial relationship between positive *ITGAM* expression and AML classification and clinical features, we further analyzed the pathway enrichment in the high versus low *ITGAM* expression group using TCGA-LAML expression profile data. The results show that highly expressed *ITGAM* is mainly enriched in cytokine-cytokine receptor interactions, cell adhesion molecules (CAMs), lysosomes, and hematopoietic cell lineages, while a large number of highly expressed *ITGAM* group genes are involved in immune infiltration and inflammation-related signaling pathways, such as Intestinal immune network for IgA production, antigen processing and presentation, Fc gamma R-mediated phagocytosis, Toll-like receptor signaling pathway, NOD-like receptor signaling pathway, Natural killer cell-mediated cytotoxicity, MAPK signaling pathway, Fc epsilon RI signaling pathway, and TGF-beta signaling pathway, etc. (Figure 11).

### Association of prognostic genes expression levels with immune score and stroma score in TCGA-LAML cohort

**Figure 12:**
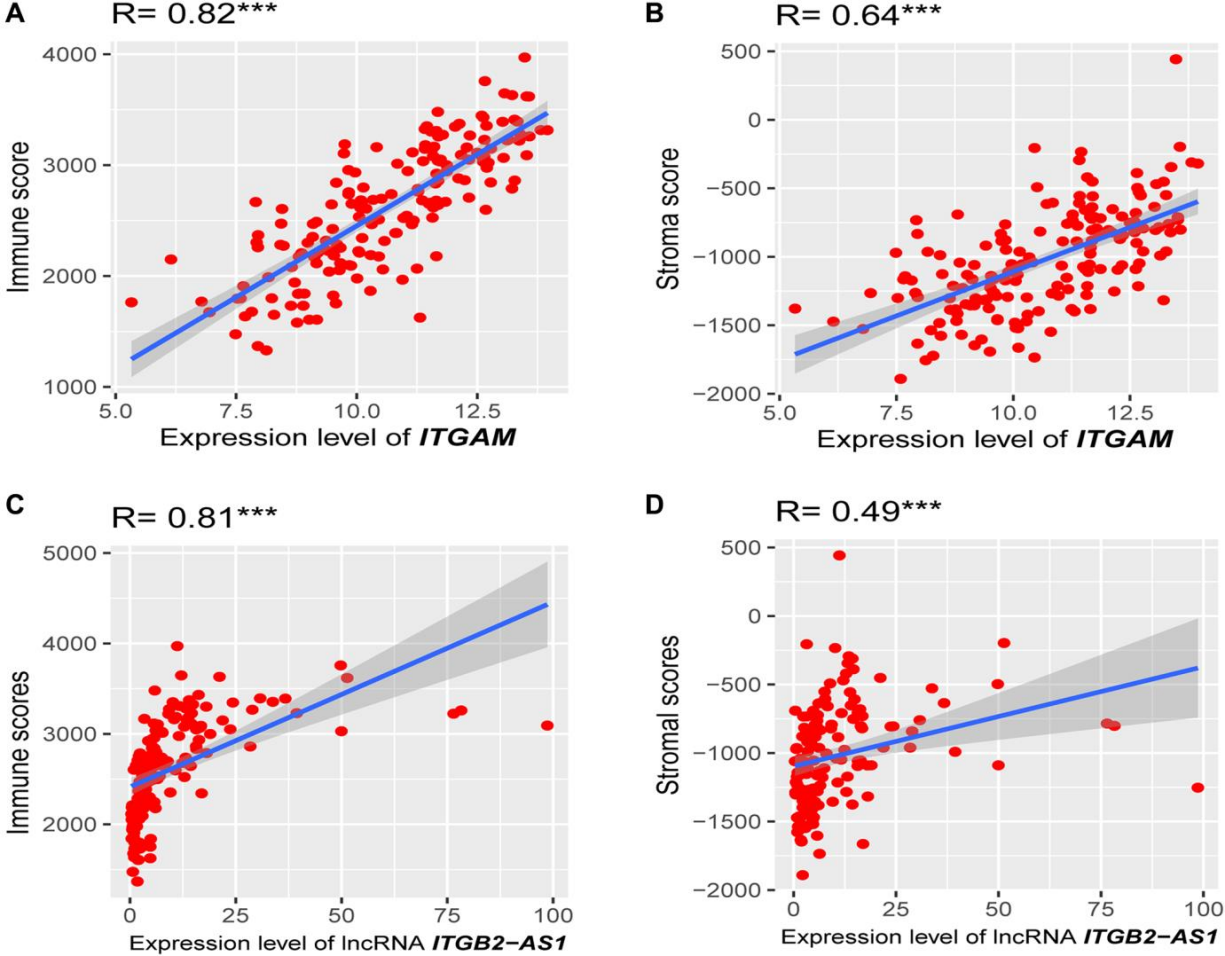
**Association of prognostic genes expression levels with the tumor microenvironment (TME).** (**A**) Strong positive correlation between *ITGAM* expression (log2 transformation) and immune score. (**B**) A moderate correlation between *ITGAM* expression (log2 transformation) and stroma score. (**C**) Strong positive correlation between LncRNA *ITGB2-AS1* expression (log2 transformation) and immune score. (**D**) A moderate correlation between LncRNA *ITGB2-AS1* expression (log2 transformation) and stroma score. *R*, Spearman’s correlation coefficient; ^***^P < 0.001.

Since the highly expressed *ITGAM, PPBP,* and LncRNA *ITGB2-AS1* are highly expressed in our internal cohort, we investigated the association of these three prognostic genes expression levels with immune score and stroma score. Interestingly, we found that the expression level (log2 transformation) of LncRNA *ITGB2-AS1* was strongly correlated with the immune score (Spearman's correlation test, *R* = 0.81, *P* < 0.001) and moderately correlated with stroma score (Spearman's correlation test, *R* = 0.49, *P* < 0.001) (Figure 12A and 12B). Besides, the expression level (log2 transformation) of *ITGAM* was also strongly correlated with the immune score (Spearman's correlation test, *R* = 0.82, *P* < 0.001) and moderately correlated with the stroma score (Spearman's correlation test, *R* = 0.64, *P* < 0.001) (Figure 12C and 12D). However, we did not find significant correlations between the expression level of *PPBP* and immune scores and stromal scores in the TCGA-LAML cohort (*R* < 0.30, *P* < 0.01). This result indicated that the expression levels of *ITGB2-AS1 and ITGAM* are associated with the modulation of immune and stromal activity in LAML.

### Association of *ITGAM* and *LncRNA ITGB2-AS1* expression levels with immune infiltrations in the TCGA-LAML cohort

**Figure 13:**
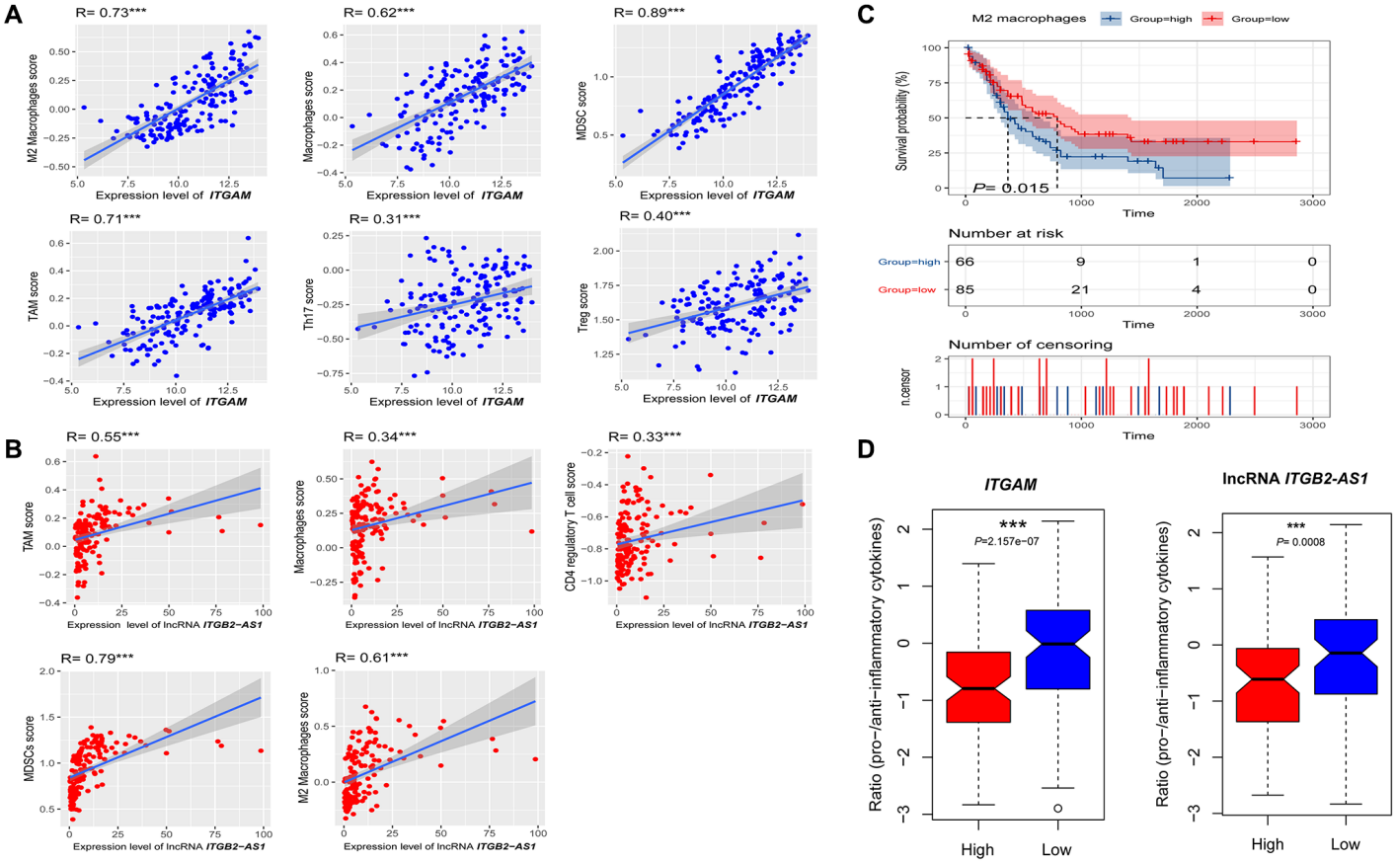
**Association of *ITGAM* and LncRNA *ITGB2-AS1* expression level with immune signature in AML.** (**A**) The expression of *ITGAM* exhibit a significant positive correlation with six immune cells (M2 Macrophages, Macrophages, Treg, MDSC, TAM, and Thr17). The Spearman's correlation test *P* values are shown; (**B**) The expression of LncRNA *ITGB2-AS1* exhibit a significant positive correlation with five immune cells (M2 Macrophages, Macrophages, MDSC, TAM, and CD4 regulatory T cells). The Spearman's correlation test *P* values are shown; (**C**) High infiltration levels (ssGSEA scores) of M2 macrophages associated with shorter survival time in LAML patients. (**D**) the ratios of pro-/anti-inflammatory cytokines are significantly lower in AML with highly expressing of *ITGAM* and LncRNA *ITGB2-AS1* (expression levels > median) than in those lowly expressing of *ITGAM* and LncRNA *ITGB2-AS1* (expression levels < median). The mean expression (log2 transformed) ratio of the marker genes levels was defined as pro-inflammatory cytokines representing immune-stimulatory signature with marker genes as IFN-γ, IL-1, and IL-2. The anti-inflammatory cytokines represent with immune-inhibitory signature with marker genes as TGFB1, IL-10, IL-4, and IL-11. Abbreviations: Treg: The regulatory T cells, TAM: Tumour-associated macrophages, MDSC: Myeloid-derived suppressor cells, TGFB: transforming growth factor–β1. ^***^P < 0.001.

Since *ITGAM* and LncRNA *ITGB2-AS1* expression levels are correlated with immune and stromal scores in the TCGA-LAML cohort, we investigated the correlations of *ITGAM* and LncRNA *ITGB2-AS1* with the various immune signatures. We revealed significant positive correlations of the *ITGAM* expression levels with the enrichment levels (ssGSEA scores) of immune inhibitory cells, including Tregs, Th17, MDSC, macrophages, TAM, and M2 macrophages (Spearman's correlation test, *P* < 0.001) (Figure 13A) but not correlated with other immune cells including B cell, CD8+ T cells, CD4+ regulatory T cells, NK cells, and CAFs. In addition, LncRNA *ITGB2-AS1* expression levels are correlated with the enrichment levels (ssGSEA scores) of CD4 regulatory T cells, MDSC, macrophages, TAM, and M2 macrophages (Figure 13B).

Furthermore, we investigated the survival differences between high and low infiltrated cellular groups of all positively correlated immune signatures. We found that the increased infiltration of M2 macrophage groups had a more insufficient survival time than the lower infiltration group (Figure 13C). In contrast, Tregs, Th17, MDSC, macrophages, TAM, and CD4+ regulatory T cells are not correlated with the survival differences.

Finally, the ratios of pro-/anti-inflammatory cytokines are significantly lower in AML with highly expressing of *ITGAM* and lncRNA *ITGB2-AS1* (expression levels > median) than in those lowly expressing of *ITGAM* and lncRNA *ITGB2-AS1* (expression levels < median) (*P* < 0.001) (Figure 13D).

Altogether, these results suggest that higher expression of *ITGAM* and lncRNA *ITGB2-AS1* are related to the prognosis and immune inhibition in AML. It also could explain that *ITGB2-AS1* and *ITGAM* play a specific role in immune inhibition in AML.

### Identification of candidate drugs targeting hub genes

We screened these two essential genes (*ITGAM* and *PPBP*) and co-expressed lncRNA (*ITGB2-AS1*) for drug-gene interactions by using DGIdb 4.0 database [20]. The prediction results showed that we obtained many candidate drugs that potentially target two essential genes. Still, most of the interaction types between target genes and drugs are uncertain (Table 3). Some of the immunosuppressive drugs as rovelizumab and clarithromycin had been identified as a potential medicine for AML treatment. However, as far as we know, the copper target's inhibitory effects on *PPBP* have not been tested for the treatment of AML. Our data suggest that these genes may be promising targets for developing anticancer drugs to treat patients with AML.

**Table 3:** Candidate drugs targeting essential genes.

**Target gene**	**Drug**	**Interaction types**	**Sources**	**PMIDs**
*ITGAM*	DIMETHYL SULFOXIDE	N/A	NCI	15839205
*ITGAM*	THEOPHYLLINE	N/A	NCI	9762784
*ITGAM*	LIAROZOLE	N/A	NCI	9603657
*ITGAM*	FENTANYL	N/A	NCI	15098168
*ITGAM*	PHENYLEPHRINE	N/A	NCI	10973693
*ITGAM*	MORPHINE	N/A	NCI	15098168
*ITGAM*	ATORVASTATIN	N/A	NCI	18333374
*ITGAM*	ROVELIZUMAB	Antagonist	ChemblInteractions	
*ITGAM*	CLARITHROMYCIN	N/A	NCI	12167449
*ITGAM*	HYDROCORTISONE	N/A	NCI	18028766
*PPBP*	COPPER	N/A	DrugBank	23896426

Note: The analysis results were obtained from the cancer-relevant drug-gene interactions database. Abbreviation: NCI: national cancer institute.

## DISCUSSION

AML is a group of heterogeneous hematological malignancies, and the morphology, immunology, cytogenetics, molecular biology, and clinical manifestations in AML patients were different. Recent studies have confirmed that AML is caused by multiple gene mutations involving cell proliferation, differentiation, and apoptosis, so abnormal gene expression plays a vital role in the occurrence and development of AML [21]. However, most of the abnormally expressed genes involved in the pathogenesis of AML were unclear, and their role in risk stratification and prognosis is still not fully understood [22]. To understand the biological functions of abnormally expressed genes related to adult AML patients' prognosis, we have performed a comprehensive analysis to identify the essential genes such as *ITGAM, PPBP*, and lncRNA *ITGB2-AS1* are play an indispensable role associated with the prognosis of AML.

In the present study, we performed a WGCNA on the DEGs and constructed 8 AML-related functional modules, among which the Blue module has the highest correlation with AML. GO and KEGG enrichment analyses showed that the module genes mainly involved neutrophil migration, tumor carbon metabolism, iron death, FcγR-mediated phagocytosis, HIF-1 signaling pathway, and NF-κB signaling pathway [23–25]. These results indicate that many cellular immune and inflammatory processes are nearly related to the occurrence and development of AML. Currently, the WGCNA method has been widely used to analyze large-scale datasets [6]. In this study, we used WGCNA to construct a free-scale gene co-expression network and identify highly simulated modules characterizing AML. Investigating the association between AML genomic and clinical features, we identified candidate biomarkers, which provides a practical scheme to find the characteristic biomarkers more efficiently in the complex disease pattern of AML.

Furthermore, based on the PPI network analysis, we screened out the top 10 MDGs (*S100A9, S100A8, NCF2, CYBB, ITGAM, FCER1G, FPR1, FCN1, HK3, VNN2*) in the Blue module. Also, we identified the DEmRNAs in the transcriptome further, and the top 10 core DEmRNAs as *FPR2, PPBP, ITGB2, ITGAM, APP, CEACAM8, STOM, CKAP4, ORM2, and FPR1* were identified. We found that the *ITGAM* and *FPR1* are the two common genes between MDGs and DEmRNAs, and the 7 co-expressed DElncRNAs are estimated as correlated with the essential genes. It is worth noting that the prognosis analysis results showed that AML patients with high expression of *ITGAM*, *PPBP*, and lncRNA *ITGB2-AS1* have a poor prognosis. Simultaneously, the Nomogram also indicates that the expression of these three essential genes could predict the 1, 3, and 5 years overall survival of AML. In addition, we verified these three genes' expression levels in an independent cohort of our clinical samples. We also get the results that the expression levels of these three genes in AML are significantly up-regulated. ROC analysis has shown that these three genes have an excellent diagnostic value for AML.

*ITGAM*, also known as integrin αM, *CD11* antigen-like family member B (*CD11B*), is responsible for encoding the integrin αM chain involved in monocytes' various adhesion interactions, macrophages, and granulocytes and mediates complement the ingestion process of coated particles [26, 27]. It has been found that the expression of *ITGAM* in AML is associated with an unfavorable prognosis [28]. Many studies have reported that *ITGAM* could be a cell surface receptor selectively expressed in leukocytes, and positive expression of *ITGAM* could predict a poor prognosis for AML patients [29]. It also plays multiple functions in the activation, chemotaxis, and cytotoxicity of the tumor microenvironment leukemia cells [19]. Current studies have found that *ITGAM* is a major non-human leukocyte antigen related to the pathogenesis of autoimmune diseases such as systemic lupus erythematosus (SLE) IgA nephropathy [27, 30, 31]. Studies have also reported that *ITGAM* is related to AML gene methylation and can be used as a differentiation marker for myeloid monocytic cell lines [32]. It can participate in bone marrow differentiation and involve in the lysine-specific demethylase 1 (LSD-1) that caused the immune escape of leukemia cells [33]. Our study further found that the expression of *ITGAM* was correlated with MDSC, Treg, TAM, M2 Macrophages, Macrophages, and Thr17. This result will provide us with a deep understanding of the immune inhibition of *ITGAM.*

Moreover, we have obtained another exciting discovery during the procession of clinical verification. When *ITGAM* expression is negative in newly-treated AML patients, the CR ratio was significantly higher than the positive group. Also, the expression of *ITGAM* could positive relative to many immune cells, which participate in the process of immune inhibition in AML. Furthermore, studies have found that the expression of ITGAM impacts AML chemotherapy resistance, and higher expression of *ITGAM/CD56* combined with lower expression of Smac/DIABLO's can be an essential predictor of AML chemotherapy resistance [34, 35]. Similarly, some studies have found that *ITGAM* has a higher prognostic value in AML patients [29]. This study found that increased expression of *ITGAM* predicted poor overall survival in AML patients and showed lower initial therapy efficacy. In addition, we grouped the TGCA-LAML samples according to the high or low expression of *ITGAM* and performed differential gene analysis and pathway enrichment analysis further, and the DEGs in the high *ITGAM* expression group were enriched in immune infiltration and inflammation-related signaling pathways. This further confirms the vital role of *ITGAM* in AML-related immune regulation and provides new insights into the role of *ITGAM* in the pathogenesis of AML.

*PPBP* (platelet photo basic protein) is the gene responsible for encoding platelet-derived growth factors. It plays a vital role in the effective chemoattractant and activation of sex granulocytes [36]. Studies have found that *PPBP* can stimulate various cellular processes, including DNA synthesis, mitosis, glycolysis, intracellular cAMP accumulation, prostaglandin E2 secretion, and hyaluronic acid synthesis sulfated glycosaminoglycans [37]. Besides, it has been confirmed that many diseases, such as essential thrombocythemia are closely related to the abnormal expression of *PPBP*. The corresponding signaling pathways include chemokines, and *NF-κB* pathways have been reported to be related to leukemia occurrence and development [38, 39]. In an mRNA analysis study of the expression profile difference between AML and healthy controls, the results showed that the expression of *PPBP* in AML patients was significantly higher than in healthy controls [40]. Another study found that *PPBP* is highly expressed in AML patients and negatively correlates with *NPM1* mutations, affecting patients' OS [38]. Studies have also shown that after the secreted protein *IGFBP7* was used to stimulate the primary CD45 cells of AML patients for 48 hours, the DNA sequencing results showed that *PPBP* was significantly high expression (*log2FC* > 2). However, it is worth considering whether these genes' abnormal expression directly affects leukemia stem cells' proliferation and differentiation. The sensitivity of chemotherapy treatment also needs further research to confirm [41]. Besides, a study found that the *PPBP* was highly expressed when the super-enhancer identified the megakaryocytes' specific epigenetic state. Still, its particular regulation. The functional mechanism remains to be further studied [42]. It should be noted that, on the contrary, current studies have also found that the expression of *PPBP* in different types of leukemia may also be significantly different, and *PPBP* may be lower-expressed in AML-M4 [43]. Therefore, the above studies only partially reveal the critical role of *PPBP* in the occurrence and development of AML. However, these vital genes' specific expression types still need to be verified in more extensive clinical studies. Their particular molecular regulation mechanisms are also worth further in-depth analysis.

During the identification and screening of co-expressed lncRNAs, we found that *ITGB2-AS1* (ITGB2 antisense RNA 1) has a significant expression correlation with the expression of critical genes. Current studies have found that *ITGB2-AS1* is closely related to the occurrence and development of various tumors, including the abnormal expression of *ITGB2-AS1* is significantly associated with astrocytoma [44]. *ITGB2-AS1* is up-regulated in osteosarcoma tissue and associated with the poor prognosis of osteosarcoma patients. The molecular mechanism studies have shown that *ITGB2-AS1* plays a vital role in osteosarcoma development and progression through Wnt/β-catenin signaling [45]. Studies have also found that *ITGB2-AS1* can increase the expression of *ITGB2* and activate integrin-related FAK signaling, thereby promoting breast cancer migration and invasion [46, 47]. Furthermore, a study has found that *ITGB2-AS1* is generally overexpressed in pancreatic ductal adenocarcinoma cell lines, and inhibiting the expression of *ITGB2-AS1* can inhibit cell proliferation, invasion, and migration process [48]. Similarly, studies based on bioinformatics analysis show that increased expression of *ITGB2-AS1* is also related to patients' poor prognosis with ovarian cancer [49].

In recent years, with the rapid development of CTLA-4, PD-1, and other immune checkpoint therapies used in AML, such as immune cells, extracellular matrix molecules, and stromal cells as critical cells in TME are getting more and more attention [50]. In our current study, to further study the molecular biological characteristics of TME in AML, we used the ESTIMATE algorithm [18] to study the immune cells closely related to the expression of *ITGAM*, *PPBP*, and lncRNA *ITGB2-AS1* in the TCGA-LAML dataset. Also, we calculated the immune score, stromal score, and ESTIMATE score for each AML sample extracted from the TCGA database by applying the ESTIMATE algorithm. The results show that the immune scores and stromal are statistically higher and are associated with the expression of *ITGAM* and lncRNA *ITGB2-AS1*. Besides, we found that the expression of *ITGAM* and lncRNA *ITGB2-AS1* are significantly positively correlated to immune inhibitory cells, including CD4+ regulatory T cells, M2 Macrophages, Macrophages, Treg, MDSC, TAM, and Thr17. Interestingly, we also found that the increased infiltration of M2 macrophage groups had a more insufficient survival time than the lower infiltration group. In contrast, Tregs, Th17, MDSC, macrophages, TAM, and CD4+ regulatory T cells are not correlated with the survival differences. These findings strongly suggest that *ITGAM* and lncRNA *ITGB2-AS1* play a specific role in prognosis and immune inhibition in LAML. Furthermore, we found that the ratios of pro-/anti-inflammatory cytokines are significantly lower in AML with highly expressing *ITGAM* and lncRNA *ITGB2-AS1* than in those lowly expressing *ITGAM* and lncRNA *ITGB2-AS1*. However, there are separate reports on the critical role of *ITGAM* in the pathogenesis of AML currently [51, 52]. Still, as we know, the research on lncRNA *ITGB2-AS1* mediated immune microenvironment in AML and its influence on the expression of pro/anti-inflammatory factors in AML is still unreported. These results indicated that *ITGAM* and lncRNA *ITGB2-AS1* could be an essential oncogene in AML, and it is likely involved in the pathogenesis of AML through involvement in the immunosuppressive TME.

Targeted therapy is a cancer treatment that uses drugs to target specific genes and proteins involved in cancer cells' growth and survival [53]. Looking for targeted drugs with anti-leukemia effect is the crucial way to treat AML [54]. This research has predicted the candidate drugs mainly targeting the critical genes in the DGIdb database. The results show that some of the immunosuppressive drugs as Rovelizumab and Clarithromycin may target the *ITGAM* gene [55, 56]. However, the pharmacological mechanism of these drugs on genes and the specific regulation mode are still unclear. It needs further research to explore the potential application value of these drugs. Similarly, as oncogenes discovered in AML, these genes' functions and mechanisms will gradually be revealed, providing new perspectives on developing and applying drugs.

Finally, this research result is still worthy of further study, and it also provides a scientific hypothesis for us. Whether the patient's positive expression with *ITGAM* and lncRNA *ITGB2-AS1* is the treatment target and whether the choice of initial treatment drugs is different due to *ITGAM* and lncRNA *ITGB2-AS1* primary expression, and these research findings and speculations will also be what we will verify in the next step.

## CONCLUSIONS

In summary, this study proves that *ITGAM, PPBP,* and *ITGB2-AS1* play an essential role in adult AML. *ITGAM* may participate in the pathogenesis of AML through AML related gene methylation and increase the immune escape of leukemia cells. *PPBP* may affect the occurrence and development of AML and the drug resistance of AML cells by participating in chemokine and *NF-κB* signaling pathways. Besides, lncRNA *ITGB2-AS1* can also be used as a potentially vital biomarker to predict the survival and risk stratification of adult AML patients. However, many prognostic-related AML-related genes, including *FLT3*, *IDH1/2*, etc., were excluded from the analysis. Therefore, the biological functions of these critical genes and lncRNAs need to identify and verify further. It is also necessary to study the physical characteristics of *ITGAM*, *PPBP,* and *ITGB2-AS1* and find their clinical application value in a larger cohort of AML patients.

## MATERIALS AND METHODS

### Acquisition and process the multi-omics data from different databases

The transcriptome profiling of GSE114868 contributed by Cheng et al. [57] was download from the GEO database (https://www.ncbi.nlm.nih.gov/geo/query/acc.cgi?acc=GSE114868). GSE114868 was a gene expression profile based on the GPL17586 platform (Affymetrix Human Transcriptome Array 2.0) containing 194 AML samples and 20 normal samples from the National Taiwan University Hospital. "Limma" [58] program in R software (version 4.0.3) was performed to background correction, normalization, and probe annotation. The DEG screening criteria were set as *FC* > 2 and *P* < 0.05. "Pheatmap" [59] and "ggrepel" [60] programs in R software (version 4.0.3) were used to draw DEGs volcano plot and heatmap.

Also, we downloaded the gene expression profiles and clinical data of 151 AML patients from the UCSC Xena [61] database (http://xena.ucsc.edu/) for further analysis. GEPIA [9] database was used to verify selected genes' effect on TCGA-LAML patients' OS. The TCGA-LAML lncRNA data was downloaded from The Cancer Genome Atlas (TCGA) database (https://portal.gdc.cancer.gov/). Furthermore, we validated the expression level of selected genes in an independent cohort based on our 20 clinical samples. We also investigated the relationship between patients' clinical characters and the expression level of a crucial gene in retrospective research based on 179 cases. Finally, we predicted the candidate drugs that target hub genes in the DGIdb 4.0 database (https://dgidb.org/).

### Construction of co-expression gene network

After we obtained the DEGs in the GSE114868 transcriptome data, R software (version 4.0.3) "WGCNA" program [62] was further used to perform a weighted gene co-expression network analysis (WGCNA). We adopted a soft threshold β to ensure the scale-free network's stability during the co-expression gene network construction. In the scale-free co-expression network, genes with high correlation were clustered in the same module. The hierarchical clustering of the weighted coefficient adjacency matrix was used to identify the functional modules. The topological overlap matrix (TOM) has been calculated further.

### The PPI network analysis and identification of crucial module driver genes

After performing WGCNA on the transcriptome data, we established a total of 8 AML related dysfunctional modules. The R software (version 4.0.3) "ClusterProfiler" program [63] was used to annotate the GO function of DEGs in the Blue module. At the same time, we used Cytoscape software (version 3.8.0) "ClueGO" program [64] to perform KEGG pathway enrichment analysis in Blue module genes. Besides, we also used the STRING database [65] to analyze the PPI in Blue module genes; the interaction network was constructed by interaction relationship score > 0.9. Finally, we used Cytoscape software (version 3.8.0) "MCODE" program [66] to screen for the MDGs, and the top 10 module genes with the highest scores were defined as MDGs. The PPI network and MDRs were both visualized in Cytoscape software (version 3.8.0).

### Screening of core differentially expressed mRNAs in whole transcriptome and identification of co-expressed differentially expressed lncRNAs

To further verify the DEmRNAs and co-expressed lncRNAs in the transcriptome. We firstly screened the DElncRNAs and using R software (version 4.0.3) "pheatmap" [59] programs to draw the heatmap of DElncRNAs. Secondly, we used more stringent screening criteria (|logFC ≥ 2|, *P* < 0.05) to identify DEmRNAs, and perform PPI analysis in the STRING [65] database (score > 0.9). Then, we used Cytoscape software (version 3.8.0) "cytoHubba" [67] program screened the core DEmRNAs, and the top 10 DEmRNAs with the highest contribution (Degree) were defined as the core DEmRNAs in the whole transcriptome. The Cytoscape (version 3.8.0) displays the PPI network and the regulatory network of core DEmRNAs across the entire transcriptome. Finally, the Person correlation coefficient (PCC) [68] was used to analyze the correlation between core DEmRNAs and DElncRNAs. The DElncRNAs-mRNAs pair with a strong correlation (PCC > 0.8, *P* < 0.05) were selected for the further two-way clustering, the R software (version 4.0.3) "corrplot" [69] program performed to draws a heatmap of the expression correlation between the core DEmRNAs and DElncRNAs.

### Prognostic analysis of critical genes in TCGA-LAML

To further verify the prognostic value of both the MDGs, core DEmRNAs, and co-expressed DElncRNAs in AML, we used the GEPIA database to perform clinical verification of TCGA-LAML patients' overall survival (OS) for these critical genes. Moreover, by using the downloaded clinical data of TCGA-LAML from the UCSC Xena [61] database and the "survival" [70] and "nomogramEx" [71] programs in R software (version 4.0.3), we have analyzed the univariate Cox risk proportional model and the Nomogram respectively. Survival analysis uses the Log-rank test for hypothesis testing. A Cox proportional hazard model was used to estimate the essential genes' hazard ratio and 95% confidence interval. *P* < 0.05 is considered statistically significant.

### The ROC curve analysis of the diagnostic value in core DEmRNAs and co-expressed DElncRNAs

Only to verify the diagnostic value of core DEmRNAs and co-expressed DElncRNAs in AML. ROC curve analysis was used for these genes to determine the AUC. AUC ≤ 0.50 indicates no, 0.8 ≤ AUC ≤ 1.0 shows good diagnostic value. Statistical analysis and visualization are based on R software (version 4.0.3) "ROCR" [72] program.

### qRT-PCR validated the expression of critical genes in initial diagnosis patients with AML

To further increase the research results' reliability, we verified the expression level of three essential genes in bone marrow mononuclear cells of newly diagnosed AML patients (WHO-AML criteria confirmed the diagnosis [73], cases with AML-M3 were excluded) by qPCR. The bone marrow samples were collected in heparinized tubes before treatment and shipped to the laboratory within 24 to 36 hours. The leukemic cells were isolated by density gradient centrifugation using 1.077 g/mL Ficoll-Isopaque (Pharmacia). The proportion of leukemic cells was estimated using May-Grünwald- Giemsa-stained cytocentrifuge preparations and light microscopy. The cell samples selected for analysis contained at least 90% blasts after separation. Pellets of 2 to 10 million cells were stored in TRIzol (Invitrogen, Carlsbad, CA, USA) and immediately frozen at –80°C. Peripheral blood mononuclear cells from 15 anonymized healthy volunteers were included as control samples.

cDNA was generated using the Reverse Transcription Kit (Foregene, Chengdu, China). The expression levels of *ITGAM*, *PPBP,* and *ITGB2-AS1* were quantified using SYBR Green Master Mix (SYBR GREEN, Beijing, China), and the housekeeping gene GAPDH was used as an internal control. The following primers used in Table 4. qRT-PCR performed on ViiATM 7 System software (Thermo Fisher Scientific, ABI7500, USA). The results were normalized to the expression of GAPDH and presented as the fold change (2^−ΔΔCT^).

**Table 4:** The primers for qRT-PCR.

**Target**	**Sequence (5′–3′)**
ITGB2-AS (human)-RT-F	AAGGCAGGTGAGTGTAGGAAGGAG
ITGB2-AS (human)-RT-R	GGAAGGCAGAGGAGGGAGGAAC
ITGAM (human)-RT-F	CTGTTTACCTGTTTCACGGAAC
ITGAM (human)-RT-R	GATTGCCTTGACTCTCAGTACT
PPBP (human)-RT-F	AGACAGTGACTTGTATGCTGAA
PPBP (human)-RT-R	TTTCTTGATTCTGGGAGCATCT
GAPDH (human)-RT-F	AGAAGGCTGGGGCTCATTTG
GAPDH (human)-RT-R	AGGGGCCATCCACAGTCTTC

### Evaluation of the impact of critical genes on patient's clinical characteristics through a retrospective study

Also, to further study the effect of *ITGAM* on the patient's clinical characteristics with AML, we have retrospectively investigated 179 cases of newly diagnosed with AML admitted to the First Affiliated Hospital of Xinjiang Medical University from March 2019 to September 2020. The patients included in this study all received Immunophenotyping tests according to WHO diagnostic criteria. The test process followed the manufacturer's indication as these steps: we obtained 1–2 ml bone marrow from patients with initial diagnosed AML and collecting in an anticoagulated tube (1–10 × 10^5^ cells/ml). Mixed the bone marrow (50μL) with fluorescein (*FITC, PE, PerCP*, and *APC*; BD Bioscience, USA) labeled antibodies (5–20 μL). Incubate for 15 minutes without light at room temperature. Add 1ml hemolysin at room temperature for 10 minutes without light. Centrifuge at 1500 rpm for 5 minutes, then discarded the supernatant. After that, the precipitation was washed twice with PBS, and the cells were analyzed using a flow cytometer (FACSCalibur, BD Bioscience, USA).

Finally, we analyzed the relationship between the expression of these essential genes and the clinical characteristics and chemotherapy efficacy of AML patients. The outcomes of chemotherapy presented as CR, PR, NR, and death. The statistical analyses used GraphPad Prism 8.0 software (CA, USA) as appropriate. Comparing patient characteristics and chemotherapy outcomes between *ITGAM* positive and negative groups adopts Student *t* and Chi-square test. The features with *P* < 0.05 were considered a significant difference between the two groups.

### Pathway analysis based on grouping *ITGAM* with high and low expression in TCGA-LAML

To explore the pathway enrichment after grouping *ITGAM* with high and low expression, we downloaded the expression profile files of AML samples (*n* = 151) from the TCGA database and performed DEGs analysis after grouping them with the median *ITGAM* expression. Then the GSEA [74] analysis was used to identify the enriched KEGG pathways, and the significant pathways were confirmed as an enrichment when FDR < 0.05.

### Evaluation of the ESTIMATE scores and immune signature enrichment levels

ESTIMATE is an algorithmic tool based on the R package for predicting tumor purity, which uses the gene expression profiles of 141 immune genes and 141 stromal genes to generate ESTIMATE scores, immune scores, and stromal scores [18]. The presence of infiltrated immune cells and stromal cells in tumor tissues were calculated using related gene expression matrix data, represented by immune and stromal scores.

We also identified the immune signature's enrichment level in the TCGA-LAML sample as the single-sample gene-set enrichment analysis (ssGSEA) score [75]. The gene set contains the collection of all marker genes of an immune signature. We included 11 immune signatures: B cell, CD8+ T cells, CD4+ regulatory T cells, NK cells, Tregs, MDSC, TAM, macrophages, M2 macrophages, CAFs, and Th17. The threshold is the absolute value of *R* is not less than 0.30 and *P* < 0.01. The marker genes set of individual immune signatures displayed in Supplementary Table 1.

Furthermore, we used the Wilcoxon test to compare the ratio of pro-inflammatory cytokines and anti-inflammatory cytokines between the TCGA-LAML with high expression levels of the *ITGAM* and lncRNA *ITGB2-AS1* (expression levels > median) and the TCGA-LAML with low expression levels of the *ITGAM* and lncRNA *ITGB2-AS1* (expression levels < median). Besides, the ratio of mean expression (log2 transformed) of the marker genes levels defined as the ratio of pro-inflammatory cytokines (IFN-γ, IL-1, IL-2) and anti-inflammatory cytokines (TGFB1, IL-10, IL-4, IL-11) in AML [76].

In addition, to investigate the survival differences between high and low infiltrated cellular groups of all positively correlated immune signatures, the immune cells infiltrate data, and the TCGA-LAML survival data (*n* = 151) were used in the K-M survival analysis, "survival" programs [70] in R software (version 4.0.3) were used to analyze and visualize the results.

### Prediction of candidate drugs target on essential genes

We identified the drugs that target the hub genes using DGIdb 4.0 [20]. DGIdb collects drug-gene interaction data from 30 disparate sources, including ChEMBL, DrugBank, Ensembl, NCBI Entrez, PharmGKB, PubChem, Clinical Trial Databases, and literature in NCBI PubMed. The drug-gene interactions supported by at least one database or PubMed reference were identified. From the identified drug-gene interactions, we selected the drugs that have been shown in the DGIdb database.

### Ethics approval and consent to participate

All research was conducted with integrity and in line with generally accepted ethical principles and approved by the Research Ethics Committee of the First Affiliated Hospital of Xinjiang Medical University (reference number: 220200320). The study was performed following the principles of the Declaration of Helsinki. All of the participants provided written informed consent to participate in this study. The ethics mentioned above committee approved this consent procedure. All personal information relating to the samples involved in the study was anonymized.

## Supplementary Materials

Supplementary Table 1
