# Supplementary material for: Identification and validation of inferior prognostic genes associated with immune signatures and chemotherapy outcome in acute myeloid leukemia
Source: Aging (Albany NY). 2021 Jun 18;13(12):16445–70. doi: 10.18632/aging.203166 (PMC8266366; doi:10.18632/aging.203166)
Supplement: Supplementary Table 1 [file aging-13-203166-s001.pdf]

SUPPLEMENTARY TABLE

Supplementary Table 1. The marker genes set of individual immune signatures.

B cells	CD4+ Regulatory T cells	CD8+ T cells	Macrophages	NK cells	Treg genes	TAM	M2 Macrophage	CAFs	Th17	MDSC
60468	400359	925	219833	3821	3595	6347	51338	1674	23261	64098
55024	1493		968	51348	164656	968	11326	59	132014	3683
640	50943		23601		1075	3586	9332	2191	286676	5732
151888	2838		1536		55353			3371	19	80142
973	9235		2517		7037			7431	84818	6279
974	3565		10457		153830			1293	23765	5660
115350	3567		9956		81831			1464	27189	83706
115352			5641		134637			1278	3605	63940
84329			4318		9435			1292	85478	6280
55103			116211		1493			1291	286	3684
					9603			10631	51561	942
					51474			1277	284948	10068
					7850			1634	112744	2213
					29851			7070	79836	7852
					84263			5159	81792	3566
					10553			5156	1084	729230
					2280			6275	79155	914
					201633				57560	2212
					1237				8707	2214
					4049				5243	929
					54733				958	
					50615				10803	
					10768				118932	
					8835				388125	
					51513				145741	
					598				760	
					10325					
					2182					
					8973					
					10538					
					54900					
					141					
					7293					
					55608					
					29126					
					834					
					4065					
					27020					
					6753					
					2926					
					1439					
					55751					

54602
253461
90459
7187
4664
9953
143903
3716
7421
54741
2650
5795
22807
1435
953
8784
25840
8844
54434
23705
3554
54
56548
63892
57126
10725
8427
28986
